# Retrospective analysis of CD4 count trends in South Africa

**DOI:** 10.4102/sajhivmed.v25i1.1651

**Published:** 2024-12-19

**Authors:** Naseem Cassim, Lindi-Marie Coetzee, Manuel P. da Silva, Deborah K. Glencross, Wendy S. Stevens

**Affiliations:** 1 Wits Diagnostic Innovation Hub, Faculty of Health Sciences, University of the Witwatersrand, Johannesburg, South Africa; 2 National Priority Programme, National Health Laboratory Service, Johannesburg, South Africa; 3 Faculty of Health Sciences, University of the Witwatersrand, Johannesburg, South Africa

**Keywords:** HIV, CD4, advanced HIV disease, laboratory data, data repository

## Abstract

**Background:**

South Africa has the largest HIV epidemic globally. Despite the scale-up of antiretroviral therapy, people living with HIV are still presenting with low CD4 counts.

**Objectives:**

This study assessed CD4 trends.

**Method:**

A retrospective analysis of laboratory data from 2013 to 2023 was conducted. Annual test volumes, the median CD4, and the percentage of specimens with a count ≤ 200 cells/µL and > 500 cells/µL were reported at the national and provincial levels, and by age and gender. The percentage change in both CD4 categories between 2013 and 2023 was assessed, and the CD4 counts per 100 000 population reported.

**Results:**

Data are reported for 32 154 644 specimens. The overall median CD4 increased from 396 cells/µL to 473 cells/µL. The percentage of specimens with CD4 counts > 500 cells/µL increased over time but the percentage with CD4 counts ≤ 200 cells/µL remained stable. Men had lower CD4 median and higher percentage of specimens with counts ≤ 200 cells/µL than women. However, the rate of, CD4 ≤ 200 cells/µL decreased from 1411 to 700 per 100 000 population; this decrease occurred in all provinces except the Western Cape.

**Conclusion:**

This study found high percentage of specimens with CD4 counts ≤ 200 cells/µL despite an increase in median CD4 count. Men had lower CD4 counts than women.

**What this study adds:** The percentage of specimens with CD4 counts ≤ 200 cells/µL remained stable over time but the rate of CD4 counts ≤ 200 cells/µL declined. Men had lower CD4 counts than women.

## Introduction

South Africa has the largest HIV epidemic in the world, with 7.7 million people living with HIV (PLHIV) in 2023.^1^ Of these, 77% of PLHIV are on antiretroviral therapy (ART).^1^ PLHIV are eligible for ART irrespective of age, CD4 count and clinical stage, and should start treatment within 7 days in the absence of clinical contraindications.^2^ Laboratory services are essential in establishing baseline clinical evaluations, including HIV status confirmation and screening for opportunistic infections such as tuberculosis and cryptococcal disease.^2^ Patients enrolled on ART are monitored using HIV viral load rather than annual CD4 testing, as per 2023 guidelines.^3^ Additional CD4 testing is only indicated for patients on cotrimoxazole preventive therapy and those with an HIV viral load ≥ 1000 copies/mL.^2,3^

Since the initial rollout of CD4 testing in 2004, several local (Southern African HIV Clinician Society and National Department of Health) and international (World Health Organization [WHO]) guideline changes have been implemented.^2,3,4,5,6,7,8^ The 2004 guidelines recommended ART initiation based on a CD4 count < 200 cells/µL and clinical manifestation of stage III/IV HIV disease.^4,9^ Later guidelines increased the CD4 threshold for ART initiation to 350 cells/µL in 2009 and then to 500 cells/µL in 2013.^6,10^ A major shift was made with the 2016 guidelines, recommending that PLHIV be initiated on ART regardless of CD4 count, that is, Universal Test and Treat (UTT).^7,11^ Baseline CD4 monitoring was, however, retained for identifying patients at highest risk of opportunistic infections. Cryptococcal disease is confirmed with a laboratory reflexed cryptococcal antigen (CrAg) screening test in patients with a CD4 ≤ 100 cells/µL, while ART prioritisation of patients with a CD4 ≤ 350 cells/µL and fast-tracking CD4 ≤ 200 cells/µL continued.^5^ The latest 2023 guidelines provided similar recommendations for CD4 and CrAg reflex testing.^3^ The latter was introduced nationally in South Africa from June 2017.^12,13^

Diagnostic services in the public sector in South Africa are provided through a network of testing laboratories operated by the National Health Laboratory Service (NHLS).^14^ Following the operational plan for HIV, laboratory services scaled up dramatically to increase both diagnostic testing and monitoring of patient safety from 2004 onwards, specifically for CD4,^15^ and later for HIV viral load.^16,17^ CD4 testing in the NHLS follows an integrated tiered service delivery model that extends coverage and improves turn-around time in a cost-effective manner.^18,19^ In addition, services have been extended from academic centres to district and community laboratories to address coverage gaps and the increased demand for services.^18,20,21^

Despite the introduction of UTT, up to half of PLHIV present with advanced HIV disease (AHD), defined for adults and adolescents as a CD4 cell count < 200 cells/µL or a WHO Stage III and IV defining illness.^22,23^ The WHO 2017 guidelines recommended that a package of screening, prophylaxis, rapid ART initiation, and intensified adherence interventions be offered to PLHIV presenting with AHD.^23^

Rates of morbidity and mortality among PLHIV are lowest in those with a CD4 ≥ 350 cells/µL in comparison to those with a CD4 < 200 cells/µL.^24^ Local normal CD4 count reference ranges reported for HIV-negative individuals were ≥ 500 cells/µL (503–2051).^25^ PLHIV newly eligible for ART with baseline CD4 counts ≥ 500 cells/µL are reported to have better outcomes than those initiating ART with lower counts.^26^ As such, PLHIV with a CD4 count > 500 cells/µL could be indicative of patients doing well on treatment or presenting early for treatment initiation, before their immune systems are compromised.

Comparative CD4 data analysis for selected years (2010/11 and 2014/15), across 52 South African districts, showed a marked decrease, where > 10% of all specimens tested had a count < 100 cells/µL.^27^ Additional data using CD4 outcomes for the last 10 years (2012–2022) were recently reported locally.^28^ This study showed that even with a 6% decline in CD4 test volumes between 2013 and 2022, the reduction in specimens with a count ≤ 200 cells/µL was only 2.8%, with a larger percentage reporting a count > 500 cells/µL.^28^ The NHLS data warehouse has collective CD4 specimen data from 2013 to date that can be analysed to provide important programmatic insights; highlighting trends and identifying geographical regions with higher percentage of specimens with CD4 counts ≤ 200 cells/µL than reported nationally.^27,29,30^

### Objective

The objective of this study was to describe trends in CD4 counts ≤ 200 cells/µL and > 500 cells/µL between 2013 and 2023 at the national and provincial levels in South Africa.

## Research methods and design

### Context

CD4 testing for the study period was offered using the following platforms: (1) XL-MCL, (2) FC500 MPL/CellMek and (3) Aquios CL cytometers. These platforms were supplied by Beckman Coulter (Miami, Florida, United States) as per the national tender agreements between 2004 and 2023.^31,32^ Irrespective of the instruments placed during the test period, a standardised CD4 PanLeucoGating protocol was used.^31^ Given the absence of a national unique patient identifier, the number of specimens reported will not correlate to the number of patients. The de-duplication of laboratory data is possible using a probabilistic matching algorithm. However, data from 2004 to 2012 are not available for this iteration, making it difficult to de-duplicate data.

### Study design

The retrospective analysis of laboratory data was conducted for the period between 01 January 2013 and 31 December 2023.

### Data preparation

The data extract was provided by the NHLS laboratory data repository and included the following variables: (1) episode number, (2) result authorisation date, (3) age (in years), (4) gender, (5) province, (6) health district, (7) testing laboratory, and (8) absolute CD4 count. The age and gender are provided on the request form and captured on the laboratory information system. The year and month were extracted from the result authorisation date. The absolute CD4 count was categorised as < 100 cells/µL, ≥ 100 – ≤ 200 cells/µL, > 200 – ≤ 350 cells/µL, > 350 – ≤ 500 cells/µL, and > 500 cells/µL. For detailed analysis, predominantly data for CD4 counts ≤ 200 cells/µL and > 500 cells/µL are reported. Analysis of all CD4 categories across the test period at a national and provincial level is reported as supplementary data (Online Appendix 1). Age was categorised as follows: ≤ 5, 6 – 10, 11 – 14, 15 – 24, 25 – 34, 35 – 44, 45 – 54, ≥ 55, or unknown. Gender was labelled as male, female, or unknown. Age and gender results are only reported at the national level in this article. The data sets were prepared and analysed using SAS 9.4 (SAS Institute, Cary, North Carolina, United States) and Stata SE (StataCorp LLC, College Station, Texas, United States).

### Statistical analysis

The annual number of specimens tested as well as the median CD4 (with interquartile ranges [IQRs]) were reported for all ages and for those ≥ 15 years. The percentage change in the year-on-year number of specimens tested was also assessed. The number of specimens tested and the percentage of specimens with a count of ≤ 200 cells/µL and > 500 cells/µL were reported at the national and provincial levels, as well as by age and gender. The provincial analysis by calendar year assessed the percentage of specimens categorised as ≤ 200 cells/µL and > 500 cells/µL, with the percentage change between 2013 and 2023 indicated. Conditional formatting was used with inverse rules applied for a count ≤ 200 cells/µL compared to > 500 cells/µL. The provincial population mid-year estimates were used to report the percentage of specimens categorised with a count ≤ 200 cells/µL per 100 000 population for 2013 and 2023, with the change over time indicated in Equation 1:

$$\Delta t=\left(\frac{CD4\leq 200}{Provincial\;Population}\times 100000\right)$$ [Eqn 1]

### Ethical considerations

Ethical clearance to conduct this study was obtained from the University of the Witwatersrand Human Research Ethics Committee (Medical) (reference no.: M220163). Anonymised secondary laboratory data were used.

## Results

### National analysis

Data are reported for 32 154 644 CD4 specimens, with the annual volumes decreasing from 3 783 437 in 2014 to 2 135 648 by 2023 (Table 1). The biggest decrease was noted between 2019 and 2020, at 13.4%. Between 2014 and 2019, the percentage change for the year-on-year number of specimens tested ranged from −0.4% (2019) to −9.5% (2017). The overall median CD4 ranged from 396 cells/µL (IQR: 233–581) to 473 cells/µL (IQR: 248–716). The percentage of specimens with a CD4 ≤ 200 cells/µL was 20.3% overall, ranging from 19.5% (2020 and 2021) to 20.8% (2016 and 2017). The percentage of specimens categorised as > 500 cells/µL ranged from 34.5% (2013) to 46.9% (2022). For an age of ≥ 15 years, the annual median CD4 ranged from 387 cells/µL (IQR: 228–563) in 2013 to 467 cells/µL (IQR: 245–706) by 2022.

**TABLE 1:** Annual analysis of the number of CD4 specimens tested between 2013 and 2023, with the annual percentage change (year on year) reported.

Year	Specimens tested		YoY change (%)	≤ 200 cells/µL		> 500 cells/µL		All ages		≥ 15 years	
	*n*	%		*n*	%	*n*	%	Median	IQR	Median	IQR
2013	3 679 464	7.6		761 092	20.7	1 270 101	34.5	396	233–581	387	228–563
2014	3 783 437	7.8	2.8	749 879	19.8	1 387 421	36.7	409	242–598	401	236–581
2015	3 573 447	7.3	−5.6	729 667	20.4	1 325 780	37.1	411	237–602	403	232–586
2016	3 379 793	6.9	−5.4	702 017	20.8	1 316 260	38.9	420	236–619	412	231–604
2017	3 057 313	6.3	−9.5	635 117	20.8	1 226 483	40.1	425	235–635	417	231–621
2018	2 816 065	5.8	−7.9	569 133	20.2	1 176 300	41.8	435	241–653	427	236–640
2019	2 804 525	5.8	−0.4	571 249	20.4	1 169 726	41.7	435	239–654	427	235–641
2020	2 429 891	5.0	−13.4	472 615	19.5	1 067 302	43.9	452	249–674	444	245–663
2021	2 261 463	4.6	−6.9	440 067	19.5	1 030 118	45.6	463	251–694	456	247–684
2022	2 233 598	4.6	−1.2	446 628	20.0	1 048 210	46.9	473	248–716	467	245–706
2023	2 135 648	4.4	−4.4	435 478	20.4	982 256	46.0	465	243–706	459	240–698

**Total**	**32 154 644**	**100.0**		**6 512 942**	**20.3**	**12 999 957**	**40.4**	**428**	**240–639**	**420**	**236–625**

IQR, interquartile ranges; YoY, year on year.

Supplementary data reporting on extended CD4 categories indicated a significant decrease in CD4 values > 200 – < 500 cells/µL from 44.6% in 2013 to 33.7% in 2023 (10.9% difference), while CD4 < 200 cells/µL remained stable over time for counts 100 – 200 cells/µL and < 100 cells/µL, at around 10% each (Online Appendix 1, Figure 1-A1).

### Demographic analysis

Most of the specimens were received from patients aged 25 to 34 years (31.3%), followed by the 35 – 44 age category (28.8%). Overall, 86.4% of specimens were from those aged 15 to 54 years (Table 2). For the ≤ 5, 6 – 10 and 10 – 14 age categories, the median CD4 was 945 cells/µL, 777 cells/µL and 590 cells/µL, respectively. Excluding an unknown age and the ≤ 5, 6 – 10 and 10 – 14 age categories, the median CD4 ranged from 405 cells/µL (IQR: 217–612) for the 35 – 44 age category to 454 cells/µL (IQR: 286–649) for the 15 – 24 category. A count ≤ 200 cells/µL was reported for 7.0% of specimens for the ≤ 5 age category, 7.4% for 6 – 10, and 12.0% for 10 – 14. Excluding specimens with no age reported and ≤ 5, 6 – 10 and 10 – 14 age categories, the percentage of specimens with a CD4 ≤ 200 cells/µL ranged from 14.7% (15 – 24) to 22.9% (35 – 44). A CD4 count > 500 cells/µL was reported for 77.7% of the specimens for the ≤ 5 age category, 75.3% for 6 – 10, and 60.5% for 10 – 14. Excluding an unknown age and ≤ 5, 6 – 10 and 10 – 14 age categories, the percentage of specimens with a CD4 > 500 cells/µL ranged from 37.5% (35 – 44) to 43.2% (15 – 24). A male-to-female ratio of 0.49:1 was reported, with 63.4% of testing performed for female patients. The overall median CD4 in male patients was 136 cells/µL lower than reported for female patients. A count ≤ 200 cells/µL was reported for 29.4% of men compared to 15.9% for women. A count > 500 cells/µL was reported for 46.4% of women versus 28.7% for men.

**TABLE 2:** Demographic analysis of the number of CD4 specimens tested between 2013 and 2023, with the percentage of specimens with a count of ≤ 200 cells/µL and > 500 cells/µL reported.

Category	Specimens tested		≤ 200 cells/µL		> 500 cells/µL		Median	IQR
	*n*	%	*n*	%	*n*	%		
**Overall**	**32 154 644**	**100.0**	**6 512 942**	**20.3**	**12 999 957**	**40.4**	**428**	**240–639**
**Age category**								
≤ 5	365 037	1.1	25 652	7.0	283 796	77.7	945	546–1413
6 – 10	447 759	1.4	33 295	7.4	337 038	75.3	777	504–1078
11 – 14	447 478	1.4	53 521	12.0	270 536	60.5	590	365–830
15 – 24	3 552 512	11.0	523 474	14.7	1534 042	43.2	454	286–649
25 – 34	10 055 740	31.3	2 025 960	20.1	3 987 487	39.7	424	240–628
35 – 44	9 260 065	28.8	2 122 958	22.9	3 474 014	37.5	405	217–612
45 – 54	4 898 213	15.2	1 057 301	21.6	1 901 300	38.8	415	228–625
≥ 55	2 503 207	7.8	497 184	19.9	995 219	39.8	424	242–633
Unknown	624 633	1.9	173 597	27.8	216 525	34.7	375	177–596
**Gender**								
Female	20 373 796	63.4	3 234 172	15.9	9 452 283	46.4	474	284–684
Male	9 978 050	31.0	2 931 734	29.4	2 864 345	28.7	338	171–536
Unknown	1 802 798	5.6	347 036	19.2	683 329	37.9	417	246–609

IQR, interquartile range.

### Provincial analysis

Most of the testing was performed in the KwaZulu-Natal (32.0%), Gauteng (21.0%), and Eastern Cape (10.9%) provinces (Table 3). The overall median CD4 ranged from 389 cells/µL (IQR: 207–599) for Gauteng province to 482 cells/µL (IQR: 297–691) for KwaZulu-Natal. Only the KwaZulu-Natal and Northern Cape provinces reported a median CD4 above the national value. The provincial levels of CD4 specimens with a count ≤ 200 cells/µL ranged from 14.5% (KwaZulu-Natal) to 24.2% (Gauteng). A CD4 count > 500 cells/µL was reported for between 35.6% (Gauteng) and 47.5% (KwaZulu-Natal).

**TABLE 3:** Provincial analysis of the number of CD4 specimens tested between 2013 and 2023, with the percentage of specimens with a count of ≤ 200 cells/µL and > 500 cells/µL reported.

Province	Specimens tested		≤ 200 cells/µL		> 500 cells/µL		Median	IQR
	*n*	%	*n*	%	*n*	%		
**Overall**	**32 154 644**	**100.0**	**6 512 942**	**20.3**	**12 999 957**	**40.4**	**428**	**240–639**
**Province**								
Eastern Cape	3 515 267	10.9	780 671	22.2	1 346 550	38.3	410	223–623
Free State	1 686 267	5.2	376 867	22.3	620 406	36.8	401	221–607
Gauteng	6 761 201	21.0	1 636 177	24.2	2 406 757	35.6	389	207–599
KwaZulu-Natal	10 304 286	32.0	1 493 496	14.5	4 894 299	47.5	482	297–691
Limpopo	2 196 534	6.8	524 978	23.9	823 945	37.5	401	209–620
Mpumalanga	2 908 127	9.0	603 898	20.8	1 132 504	38.9	417	234–627
North West	2 012 037	6.3	474 919	23.6	739 758	36.8	397	211–611
Northern Cape	685 679	2.1	141 150	20.6	281 693	41.4	431	238–649
Western Cape	2 085 246	6.5	480 786	23.1	754 045	36.2	396	215–601

IQR, interquartile range.

Supplementary data reporting on extended CD4 categories at the provincial level showed that the percentage of specimens with a count < 100 cells/µL at the provincial level ranged from 6.4% (KwaZulu-Natal) to 12.3% (Limpopo) (Online Appendix 1, Figure 2-A1). The Western Cape province reported the highest percentage of specimens with a count ≥ 100 – ≤ 200 cells/µL (12.3%) compared to 8.1% for KwaZulu-Natal. For CD4 > 200 – ≤ 500 cells/µL, contributions ranged from 38.0% (KwaZulu-Natal) to 40.9% (Free State).

### Provincial analysis by year

For 2013, the provincial percentage of specimens with a count ≤ 200 cells/µL ranged from 16.8% (Western Cape) to 24.1% (Limpopo) (Table 4). Between 2013 and 2023, there was a 4.8% reduction in specimens with a CD4 ≤ 200 cells/µL in KwaZulu-Natal compared to a 10.8% increase in the Western Cape. A 3% increase was reported for the Eastern Cape. For the Northern Cape, North West and Gauteng provinces, the change in a CD4 ≤ 200 cells/µL between 2013 and 2023 was < 2%. Only the KwaZulu-Natal, Limpopo and Mpumalanga provinces reported a decline in counts ≤ 200 cells/µL between 2013 and 2023. There were between 31.1% (Free State) and 37.4% (Western and Northern Cape) of specimens with a count > 500 cells/µL in 2013, with the most dramatic increase noted between 2013 and 2023 in KwaZulu-Natal (19.8%) followed by Mpumalanga (11.7%), Limpopo (11.3%), and the Free State (10.1%). The Western Cape was the only province to show a decline of 0.4% in the > 500 cells/µL group.

**TABLE 4:** Provincial analysis of the percentage of CD4 specimens with a count of ≤ 200 cells/µL and > 500 cells/µL is reported, with conditional formatting applied (green = low value and red = high value for a count ≤ 200 cells/µL, with the inverse applied for the > 500 cells/µL category).

Category	Province	CD4 specimens with a count of ≤ 200 cells/µL and > 500 cells/µL (%)											Change (%)
		2013	2014	2015	2016	2017	2018	2019	2020	2021	2022	2023	
≤ 200 cells/µL	Eastern Cape	21.4	21.0	21.8	21.4	22.1	21.7	22.4	21.9	23.6	24.6	24.4	3.0
	Free State	22.3	20.3	20.3	21.7	21.8	23.0	24.4	22.7	23.8	25.4	23.7	1.4
	Gauteng	23.6	23.1	24.5	23.8	24.7	24.4	25.1	24.4	23.6	25.0	24.8	1.2
	KwaZulu-Natal	17.7	15.9	16.1	16.2	14.8	13.9	13.2	12.2	11.8	11.6	12.9	−4.8
	Limpopo	24.1	23.2	23.8	25.4	24.5	23.3	24.1	23.4	23.4	23.6	23.4	−0.8
	Mpumalanga	20.3	21.4	21.6	22.4	21.9	21.0	21.8	19.7	18.2	18.5	18.7	−1.6
	North West	21.7	22.3	22.1	23.9	25.5	24.3	24.2	24.8	25.4	25.0	22.9	1.2
	Northern Cape	19.4	20.2	20.5	22.5	21.4	20.5	20.4	20.0	20.7	20.8	20.3	0.9
	Western Cape	16.8	16.9	18.9	21.3	23.4	25.5	27.8	27.2	27.5	28.6	27.7	10.8
> 500 cells/µL	Eastern Cape	34.1	35.6	36.1	39.0	39.2	40.6	39.6	40.9	39.9	40.2	40.3	6.2
	Free State	31.1	36.1	37.1	37.0	38.1	37.1	35.2	38.7	39.3	39.2	41.2	10.1
	Gauteng	31.8	33.8	33.4	36.9	36.1	36.4	34.8	36.3	38.8	38.8	39.3	7.4
	KwaZulu-Natal	36.9	40.2	40.8	42.9	46.8	49.5	51.3	54.3	56.7	59.3	56.6	19.8
	Limpopo	32.0	34.8	35.1	34.7	37.0	39.8	39.2	40.5	42.0	43.7	43.4	11.3
	Mpumalanga	35.9	34.0	35.0	36.3	37.7	39.9	38.6	42.2	46.0	48.2	47.7	11.7
	North West	34.3	34.9	36.9	36.7	35.4	37.6	37.3	37.0	37.1	39.1	41.9	7.6
	Northern Cape	37.4	37.0	37.8	37.4	39.8	42.8	42.4	44.4	44.6	44.8	45.6	8.2
	Western Cape	37.4	38.9	37.9	38.0	35.9	34.0	31.6	32.9	34.8	36.0	37.0	−0.4

Note: The percentage change between 2013 and 2023 is also reported. The overall national average for ≤ 200 cells/µL was 20.3%, and 40.4% for > 500 cells/µL.

When analysed with population data for 2013 and 2023, the rate of specimens classified as a CD4 ≤ 200 cells/µL per 100 000 population decreased from 1411 to 700 (Table 5). The provincial rate of specimens with a count ≤ 200 cells/µL per 100 000 population ranged from 611 in the Western Cape to 1742 in KwaZulu-Natal for 2013. Five provinces in 2013 exceeded the national value. In 2023, the provincial rate of CD4 ≤ 200 cells/µL per 100 000 population ranged from 525 in Limpopo to 876 in the Northern Cape. The biggest change in the rate of specimens with a count ≤ 200 cells/µL per 100 000 population between 2013 and 2023 was noted for KwaZulu-Natal, with a 1042 reduction, compared to an increase of 32 for the Western Cape. The overall change in population was 15.3% between 2013 and 2023, ranging from 3.5% (Eastern Cape) to 24.8% (Gauteng).

**TABLE 5:** Provincial analysis of the number of CD4 specimens with a count of ≤ 200 cells/µL expressed as a percentage and a rate per 100 000 population (change between 2013 and 2023 indicated).

Province	2013							2023							Change in ≤ 200 cells/µL per 100 000 population
	Specimens tested		≤ 200 cells/µL		Population		≤ 200 cells/µL per 100 000 population	Specimens tested		≤ 200 cells/µL		Population		≤ 200 cells/µL per 100 000 population	
	*n*	%	*n*	%	*n*	%		*n*	%	*n*	%	*n*	%		
Eastern Cape	410 634	11.2	87 904	21.4	6 912 035	12.8	1272	242 742	11.4	59 262	24.4	7 150 858	11.5	829	−443
Free State	218 451	5.9	48 714	22.3	2 831 904	5.3	1720	102 825	4.8	24 404	23.7	3 022 882	4.9	807	−913
Gauteng	820 803	22.3	193 705	23.6	12 535 773	23.2	1545	445 567	20.9	110 355	24.8	15 639 891	25.2	706	−840
KwaZulu-Natal	1 065 419	29.0	188 744	17.7	10 836 221	20.1	1742	662 297	31.0	85 283	12.9	12 161 867	19.6	701	−1041
Limpopo	274 460	7.5	66 223	24.1	5 676 599	10.5	1167	142 537	6.7	33 289	23.4	6 335 306	10.2	525	−641
Mpumalanga	350 964	9.5	71 317	20.3	4 273 804	7.9	1669	176 493	8.3	33 039	18.7	4 984 096	8.0	663	−1006
North West	246 839	6.7	53 610	21.7	3 482 672	6.5	1539	131 749	6.2	30 156	22.9	4 091 587	6.6	737	−802
Northern Cape	67 510	1.8	13 082	19.4	1 201 254	2.2	1089	58 633	2.7	11 889	20.3	1 356 580	2.2	876	−213
Western Cape	224 384	6.1	37 793	16.8	6 189 842	11.5	611	172 805	8.1	47 801	27.7	7 437 324	12.0	643	32

**Total**	**3 679 464**	**100.0**	**761 092**	**20.7**	**53 940 104**	**100.0**	**1411**	**2 135 648**	**100.0**	**435 478**	**20.4**	**62 180 391**	**100.0**	**700**	**−711**

## Discussion

This study aimed to describe trends in CD4 categories, specifically those with a count ≤ 200 cells/µL and > 500 cells/µL using laboratory outcomes over 11 years. Even though this study focused on the distribution of CD4 ≤ 200 cells/µL and > 500 cells/µL, analysis was done for extended categories (Online Appendix 1). Over time, the overall percentage contribution of a CD4 < 100 cells/µL and ≥ 100 – ≤ 200 cells/µL remained consistent, with reported ranges of 9.2% – 10.2% and 9.8% – 11.1%, respectively. The CD4 category of ≥ 200 – ≤ 500 cells/µL decreased over time, from 55.9% in 2013 to 42.9% by 2022 (Online Appendix 1, Figure 1-A1). This decrease was countered by a significant increase in count > 500 cells/µL from 34.5% (2013) to 46.9% by 2022. This is significant, as the CD4 test volumes have decreased by around 5% annually, especially since 2015, because of multiple guideline changes, with testing recommended for pre-ART as well as additional testing for patients that report a HIV viral load ≥ 1000 copies/mL.^2,3,4,5^ This also correlates with an increase in HIV viral load testing reported since 2013.^2,3,5,33^ The decrease in the number of CD4 specimens tested during the COVID-19 period has been reported locally, with confirmation in this study for 2020 specifically.^34^ What is worrying is the increase in percentage of specimens with a count ≤ 200 cells/µL in 2022 and 2023 during the post-COVID-19 period.^35^ This was confirmed by a local study that reported that despite the availability of UTT, most patients still presented late with CD4 ≤ 350 cells/µL.^36^ Another study conducted between 01 June 2014 and 31 March 2015 reported that 33% of the newly diagnosed cases presented with CD4 ≤ 200 cells/µL.^37^ A local study that estimated pooled prevalence between 2010 and 2022 showed that the burden of AHD remains high among both ART-naïve and ART-experienced patients.^38^ The findings reported here confirm that for 2023 over 20% of specimens were classified with a count ≤ 200 cells/µL, despite the dramatic uptake in ART coverage in this period (to 77%).^1^

Our findings show higher median CD4 values (> 400 cells/µL) from 2014 onwards. Overall, the median CD4 increased by 69 cells/µL between 2013 and 2023, despite the noted decline in the number of specimens tested. At the national level, the median CD4 never exceeded 500 cells/µL.^25^ However, the increase in the overall median CD4 count could potentially indicate earlier ART initiation.^38^ It has been reported that the median CD4 at ART initiation for lower- to middle-income countries ranged from 99 cells/µL (IQR:71– 140) to 234 cells/µL (IQR: 192–285), compared to between 71 cells/µL and 311 cells/µL in upper- to middle-income countries.^39^ A meta-analysis reported that the mean CD4 both at presentation and at ART initiation among adults in sub-Saharan Africa between 2002 and 2012 was 286 cells/µL.^40^ The highest mean CD4 of 370 cells/µL was reported for Uganda.^40^ Similarly, the African Cohort study reported a median CD4 of 319 cells/µL at HIV diagnosis.^41^ Recalculating the overall CD4 median excluding children < 15 years (3.9% of total tests) did not significantly change the median CD4 (428 cells/µL vs. 420 cells/µL) or IQR ranges reported at the national level.

The percentage of patients with a count > 500 cells/µL increased by 11.5% between 2013 and 2023, supporting recent work on the UTT approach.^24^ This is a promising finding that indicates that as the percentage of specimens with a count > 500 cells/µL increases, levels of virological suppression should improve.^24^ These data are supported by a local study that reported that the percentage of samples with an HIV viral load ≥ 1000 copies/mL decreased from 24.0% in 2013 to 11.6% by 2022.^32^ An analysis of laboratory data showed that the percentage of specimens with a CD4 > 500 cells/µL was 23.2% in 2010/11, rising to 36.5% by 2014/15.^25^ These findings show that the trend for a count > 500 cells/µL has exceeded earlier estimates, peaking at 46.9% by 2022.

As expected, the majority of testing was performed for those aged 15 years and older. For the younger ages T-cell subsets are recommended. As expected, the median CD4 was higher for an age below 14 years. A local study reported that assessed data between 2005 and 2011 for those 15 years and older reported that the burden of advanced disease for a first CD4 was highest for the ≥ 40, 35 – 39, and 30 – 34 age categories.^29^ In contrast, our findings show that the 35 – 39 age category reported the highest percentage of specimens, with a CD4 ≤ 200 cells/µL (excluding an unknown age). This highlights the changing patterns of a count ≤ 200 cells/µL by age categories across time. Additional analysis of the age and gender patterns should be conducted at the provincial level.

Within sub-Saharan Africa, eastern and southern Africa, specifically, ART programmes have reported gender disparities in access to testing and ART initiation.^42,43^ HIV services are predominantly focused on women of reproductive age, with HIV testing offered at various entry points that include reproductive, maternal, and child health.^42^ In contrast, men do not have equivalent entry points into HIV care.^42^ This is consistent with earlier reports for patients entering care from 2005 to 2016, where 65% were women and of reproductive age.^29^ Women contributed 65% of testing in 2017, with 76% aged 25 to 54 years.^26^ A Western Cape study that assessed population-level differences comparing men and women across the continuum of routine HIV care reported that men contributed < 35% of presented CD4 counts.^44^

Many studies reported a discrepancy in late presentation by gender.^27,29,30^ A discrepancy in the percentage of specimens with a count > 500 cells/µL by gender, that included a median difference of 148 cells/µL, is also reported in our study. This is, however, one of the first studies locally to show a difference in the group with a count > 500 cells/µL by gender compared to the group with lower CD4 counts. At a national level, there have been substantial improvements in the CD4 count between 2013 and 2023; this has not been apparent for men, who have higher percentage of specimens with a count ≤ 200 cells/µL and lower proportions with a count > 500 cells/µL. Further investigation into this phenomenon over time, as well as to identify those that require interventions to improve treatment outcomes, is required.

At the provincial level, the median CD4 was lowest in the Western Cape and highest for KwaZulu-Natal. The Gauteng and KwaZulu-Natal provinces requested over 50% of CD4 testing, which is consistent with local population data.^45^ Although this finding is consistent with an earlier study that showed KwaZulu-Natal had the lowest rate of specimens with a count ≤ 200 cells/µL, lower median CD4 in the Western Cape has not previously been reported.^46^ The analysis of the rate of specimens with a count ≤ 200 cells/µL per 100 000 population confirmed an increase in the Western Cape compared to a decrease in all other provinces. Of more concern is that between 2013 and 2023 the percentage of specimens with CD4 counts ≤ 200 cells/µL increased by 10.8% in the Western Cape, which could potentially be attributed to multiple factors. A 2020 Western Cape study reported that men presented with more advanced disease, were less likely to attend healthcare services annually or initiate ART, and had higher mortality while receiving ART.^44^ Tuberculosis and HIV coinfection could potentially also be a contributory factor for PLHIV, resulting in low CD4 counts. A local study reported that between 2012 and 2022 the change in *Mycobacterium tuberculosis* complex detection rate per 100 000 population was 380 for the Western Cape compared to 135 nationally.^47^ These findings could be contributing to a lower median CD4 noted in the Western Cape in our study. Other factors described to play a role include socioeconomic status, geospatial differences in late presentation, ART retention rate, attrition, virological suppression rates, HIV/tuberculosis coinfection, etc.^42,43,48,49^

This report demonstrates a need for better and closer monitoring of the burden of HIV disease, especially for those with a count ≤ 200 cells/µL, not only at the national level but down to the health facility level. There is also a need to extend the current results for action reports by the National Institute of Communicable Diseases developed for HIV polymerase chain reaction (positive) and HIV viral load (≥ 1000 copies/mL) to include CD4 counts ≤ 200 cells/µL.^11^ The current study also demonstrates the value of a well-curated national laboratory repository that could be used to assess trends of specimens with a count ≤ 200 cells/µL over time.^50^ The combination of laboratory and clinical data would further enrich this resource and help to elucidate how many PLHIV presented with low CD4 counts, received ART, and were virally suppressed. This has only been attempted in the Western Cape province and needs to be expanded across the country.^51^

### Limitations

The laboratory data used in this study were not able to distinguish between first-ever and follow-up CD4 tests and may account for differences in disease burden previously reported.^29^ The data may also include CD4 testing for patients already on ART, receiving immune monitoring for an HIV viral load ≥ 1000 copies/mL, in line with current guidelines.^3^ From the laboratory data, it is not possible to distinguish between CD4 testing as part of pre-ART, ART monitoring following virological failure, or interrupted treatment. The data are also not able to distinguish guidelines compliance without linking clinical data to laboratory systems. Efforts were made to de-duplicate the data in the laboratory repository; however, without specimen-level data from April 2004 onwards it is not possible to determine a first-ever CD4.^29^ It is possible that patient-level data may reveal even higher percentage of specimens with CD4 counts ≤ 200 cells/µL and a lower median CD4. Furthermore, this study only documents specimen-level data as a means to estimate the percentage of specimens with a count ≤ 200 cells/µL and likewise estimate patients with CD4 > 500 cells/µL who have effectively (re)entered the reference or normal range.^25^ This study did not investigate underlying factors that could contribute to differences in the percentage of specimens with CD4 counts ≤ 200 cells/µL. Furthermore, for those aged < 5 years, the absolute CD4 count is clinically less useful than the percentage of specimens with CD4 count. For these patients, a full T-cell subset is usually requested, as clinicians are interested in both the percentage of specimens with CD4 and a CD4:8 ratio.^52^

## Conclusion

Despite a consistent increase in the national median CD4 count since 2013, high percentage of specimens with CD4 counts ≤ 200 cells/µL continue to persist especially among men. Further, and of more notable concern, is the increase in the percentage of specimens with a count ≤ 200 cells/µL between 2013 and 2023 in the Western Cape province, which warrants urgent attention and further investigation.

Even though CD4 test numbers are declining, many patients still present with extremely low counts (immune compromised). Without CD4 testing as per HIV guidelines, together with expanded HIV viral load testing, many patients with AHD and treatment failure may go undetected. Hence, monitoring laboratory HIV viral load and CD4 data should be used as complementary tools to identify PLHIV needing additional interventions.

## References

[1] The Joint United Nations Programme on HIV/AIDS (UNAIDS). South Africa: Country factsheets [homepage on the Internet]. 2023 [cited 2024 Jul 12]. Available from: https://www.unaids.org/en/regionscountries/countries/southafrica

[2] National Department of Health (NDOH). ART clinical guidelines for the management of HIV in adults, pregnancy, adolescents, children, infants and neonates [homepage on the Internet]. Pretoria: National Department of Health (NDOH); 2019 [cited 2020 Jan 18]. Available from: https://www.knowledgehub.org.za/system/files/elibdownloads/2020-05/2019%20ART%20Guideline%2028042020%20pdf.pdf

[3] National Department of Health (NDOH). ART clinical guidelines for the management of HIV in adults, pregnancy and breastfeeding, adolescents, children, infants and neonates [homepage on the Internet]. Pretoria: National Department of Health (NDOH); 2023 [cited 2020 Jan 18]. Available from: https://knowledgehub.health.gov.za/system/files/elibdownloads/2023-07/National%20ART%20Clinical%20Guideline%20AR%204.5%2020230713%20Version%204%20WEB.pdf

[4] National Department of Health (NDOH). National antiretroviral treatment guidelines [homepage on the Internet]. Pretoria: National Department of Health (NDOH); 2004 [cited 2020 Jan 18]. Available from: https://www.gov.za/sites/default/files/gcis_document/201409/artguidelines0.pdf

[5] National Department of Health (NDOH). Implementation of the Universal Test and Treat strategy for HIV positive patients and differentiated care for stable patients [homepage on the Internet]. Pretoria: National Department of Health (NDOH); 2016 [cited 2020 Jan 18]. Available from: https://sahivsoc.org/Files/22%208%2016%20Circular%20UTT%20%20%20Decongestion%20CCMT%20Directorate.pdf

[6] World Health Organization (WHO). Consolidated guidelines on the use of antiretroviral drugs for treating and preventing HIV infection [homepage on the Internet]. 2013 [cited 2022 Aug 17]. Available from: https://www.who.int/publications/i/item/9789241505727 24716260

[7] World Health Organization (WHO). Consolidated guidelines on the use of antiretroviral drugs for treating and preventing HIV infection: Recommendations for a public health approach [homepage on the Internet]. 2016 [cited 2022 Aug 17]. Available from: https://iris.who.int/bitstream/handle/10665/208825/9789241549684_eng.pdf?sequence=1 27466667

[8] World Health Organization (WHO). Consolidated HIV guidelines for prevention, treatment, service delivery & monitoring: Recommendations for a public health approach [homepage on the Internet]. 2021 [cited 2022 Aug 17]. Available from: https://apps.who.int/iris/rest/bitstreams/1357089/retrieve 34370423

[9] Weinberg JL, Kovarik CL. The WHO clinical staging system for HIV/AIDS. Virtual Mentor. 2010;12(3):202–206. 10.1001/virtualmentor.2010.12.3.cprl1-1003 23140869

[10] World Health Organization (WHO). Rapid advice: Antiretroviral therapy for HIV infection in adults and adolescents [homepage on the Internet]. 2009 [cited 2022 Aug 17]. Available from: https://new.aidsdatahub.org/sites/default/files/resource/art-hiv-infection-adults-and-adolescents-2009.pdf

[11] Wessels J, Sherman G, Bamford L, et al. The updated South African national guideline for the prevention of mother to child transmission of communicable infections (2019). South Afr J HIV Med. 2020;21(1):1079. 10.4102/sajhivmed.v21i1.1079 32832113 PMC7433286

[12] Cassim N, Schnippel K, Coetzee LM, Glencross DK. Establishing a cost-per-result of laboratory-based, reflex Cryptococcal antigenaemia screening (CrAg) in HIV+ patients with CD4 counts less than 100 cells/μl using a Lateral Flow Assay (LFA) at a typical busy CD4 laboratory in South Africa. PLoS One. 2017;12(2):e0171675. 10.1371/journal.pone.0171675 28166254 PMC5293213

[13] Coetzee LM, Cassim N, Glencross DK. Rapid scale up of reflexed Cryptococcal antigen (CrAg) screening across a CD4 laboratory network in South Africa. International Aids Society (IAS); 2017.

[14] National Health Laboratory Service (NHLS). Annual Report 2018/19 [homepage on the Internet]. Johannesburg, South Africa: National Health Laboratory Service (NHLS); 2019 [cited 2020 Feb 19]. Available from: https://www.nhls.ac.za/key-documents/annual-reports/

[15] Glencross DK, Janossy G, Coetzee LM, et al. Large-scale affordable PanLeucogated CD4+ testing with proactive internal and external quality assessment: In support of the South African national comprehensive care, treatment and management programme for HIV and AIDS. Cytometry B Clin Cytom. 2008;74 Suppl 1:S40–S51. 10.1002/cyto.b.20384 18228554

[16] South African Government. Operational plan for comprehensive HIV and AIDS care, management and treatment for South Africa [homepage on the Internet]. 2003 [cited 2024 Feb 28]. Available from: https://www.gov.za/sites/default/files/gcis_document/201409/aidsoperationalplan10.pdf

[17] Scott LE, Noble LD, Moloi J, Erasmus L, Venter WD, Stevens W. Evaluation of the Abbott m2000 RealTime human immunodeficiency virus type 1 (HIV-1) assay for HIV load monitoring in South Africa compared to the Roche Cobas AmpliPrep-Cobas Amplicor, Roche Cobas AmpliPrep-Cobas TaqMan HIV-1, and BioMerieux NucliSENS EasyQ HIV-1 assays. J Clin Microbiol. 2009;47(7):2209–2217. 10.1128/JCM.01761-08 19420172 PMC2708481

[18] Glencross DK, Coetzee LM, Cassim N. An integrated tiered service delivery model (ITSDM) based on local CD4 testing demands can improve turn-around times and save costs whilst ensuring accessible and scalable CD4 services across a national programme. PLoS One. 2014;9(12):e114727. 10.1371/journal.pone.0114727 25490718 PMC4260875

[19] Cassim N, Coetzee LM, Schnippel K, Glencross DK. Estimating implementation and operational costs of an integrated tiered CD4 service including laboratory and point of care testing in a remote health district in South Africa. PLoS One. 2014;9(12):e115420. 10.1371/journal.pone.0115420 25517412 PMC4269438

[20] Coetzee LM, Cassim N, Glencross DK. Implementation of a new ‘community’ laboratory CD4 service in a rural health district in South Africa extends laboratory services and substantially improves local reporting turnaround time. S Afr Med J. 2015;106(1):82–87. 10.7196/SAMJ.2016.v106i1.10081 26792313

[21] Coetzee LM, Cassim N, Glencross DK. Newly implemented community CD4 service in Tshwaragano, Northern Cape province, South Africa, positively impacts result turn-around time. Afr J Lab Med. 2022;11(1):1376. 10.4102/ajlm.v11i1.1376 35811752 PMC9257740

[22] Waldrop G, Doherty M, Vitoria M, Ford N. Stable patients and patients with advanced disease: Consensus definitions to support sustained scale up of antiretroviral therapy. Trop Med Int Health. 2016;21(9):1124–1130. 10.1111/tmi.12746 27371814

[23] World Health Organization (WHO). Advanced HIV disease [homepage on the Internet]. 2017 [cited 2022 Aug 17]. Available from: https://iris.who.int/bitstream/handle/10665/255884/9789241550062-eng.pdf?sequence=1

[24] Maduna PH, Dolan M, Kondlo L, et al. Morbidity and mortality according to latest CD4+ cell count among HIV positive individuals in South Africa who enrolled in project Phidisa. PLoS One. 2015;10(4):e0121843. 10.1371/journal.pone.0121843 25856495 PMC4391777

[25] Lawrie D, Coetzee LM, Becker P, Mahlangu J, Stevens W, Glencross DK. Local reference ranges for full blood count and CD4 lymphocyte count testing. S Afr Med J. 2009;99(4):243–248. 19588777

[26] Dorward J, Sookrajh Y, Gate K, et al. HIV treatment outcomes among people with initiation CD4 counts> 500 cells/µL after implementation of treat all in South African public clinics: A retrospective cohort study. J Int AIDS Soc. 2020;23(4):e25479. 10.1002/jia2.25479 32319203 PMC7174836

[27] Coetzee LM, Cassim N, Glencross DK. Analysis of HIV disease burden by calculating the percentages of patients with CD4 counts <100 cells/microL across 52 districts reveals hot spots for intensified commitment to programmatic support. S Afr Med J. 2017;107(6):507–513. 10.7196/SAMJ.2017.v107i6.11311 28604323

[28] Coetzee L CN, Da Silva P, Stevens WS, editor. Year-on-year analysis of laboratory CD4 test results of the National Health Laboratory Service (NHLS) of South Africa: 2013 to 2022. In African Society for Laboratory Medicine (ASLM) Conference; 2023 Dec 13 December; Cape Town.

[29] Carmona S, Bor J, Nattey C, et al. Persistent high burden of advanced HIV disease among patients seeking care in South Africa’s national HIV program: Data from a nationwide laboratory cohort. Clin Infect Dis. 2018;66(suppl_2):S111–S117. 10.1093/cid/ciy045 29514238 PMC5850436

[30] Glencross DK, Cassim N, Coetzee LM. Documented higher burden of advanced and very advanced HIV disease among patients, especially men, accessing healthcare in a rapidly growing economic and industrial hub in South Africa: A call to action. S Afr Med J 2020;110:505–513. 10.7196/SAMJ.2020.v110i6.14352 32880563

[31] Coetzee LM, Glencross DK. Performance verification of the new fully automated Aquios flow cytometer PanLeucogate (PLG) platform for CD4-T-lymphocyte enumeration in South Africa. PLoS One. 2017;12(11):e0187456. 10.1371/journal.pone.0187456 29099874 PMC5669480

[32] National Health Laboratory Service (NHLS). National CD4 count testing programme [homepage on the Internet]. 2022 [cited 2022 Aug 18]. Available from: https://www.nhls.ac.za/priority-programmes/cd4/

[33] Hans L, Cassim N, Sarang S, et al. HIV viral load testing in the South African public health setting in the context of evolving ART guidelines and advances in technology, 2013–2022. Diagnostics. 2023;13(17):2731. 10.3390/diagnostics13172731 37685268 PMC10486780

[34] Madhi SA, Gray GE, Ismail N, et al. COVID-19 lockdowns in low- and middle-income countries: Success against COVID-19 at the price of greater costs. S Afr Med J. 2020;110(8):724–726. 10.7196/SAMJ.2020.v110i8.15055 32880296

[35] National Institute for Communicable Diseases (NICD). Laboratory confirmed cases of COVID-19 in South Africa [homepage on the Internet]. 2022 [cited 2022 Feb 14]. Available from: https://www.nicd.ac.za/wp-content/uploads/2022/02/COVID-19-Weekly-Epidemiology-Brief-week-5-2022.pdf

[36] Bor J, Fox M, Nattey C, editors. Late presentation persists under UTT in South Africa: A national cohort study. Boston, MA: Conference on Retroviruses and Opportunistic Infections; 2020.

[37] Fomundam H, Tesfay A, Mushipe S, et al. Prevalence and predictors of late presentation for HIV care in South Africa. S Afr Med J. 2017;107(12):1058–1064. 10.7196/SAMJ.2017.v107i12.12358 29262956 PMC5792655

[38] Kitenge M, Fatti G, Eshun-Wilson I, Aluko O, Nyasulu P. Prevalence and trends of advanced HIV disease among antiretroviral therapy-naïve and antiretroviral therapy-experienced patients in South Africa between 2010–2021: A systematic review and meta-analysis. BMC Infect Dis. 2023;23(1):549–565. 10.1186/s12879-023-08521-4 37608300 PMC10464046

[39] IeDEA T, Collaborations CC. Global trends in CD4 cell count at the start of antiretroviral therapy: Collaborative study of treatment programs. Clin Infect Dis. 2018;66(6):893–903. 10.1093/cid/cix915 29373672 PMC5848308

[40] Siedner MJ, Ng CK, Bassett IV, Katz IT, Bangsberg DR, Tsai AC. Trends in CD4 Count at Presentation to care and treatment initiation in sub-Saharan Africa, 2002–2013: A meta-analysis. Clin Infect Dis. 2014;60(7):1120–1127. 10.1093/cid/ciu1137 25516189 PMC4366582

[41] Esber AL, Coakley P, Ake JA, et al. Decreasing time to antiretroviral therapy initiation after HIV diagnosis in a clinic-based observational cohort study in four African countries. J Int AIDS Soc. 2020;23(2):e25446. 10.1002/jia2.25446 32064776 PMC7025087

[42] Cornell M, Majola M, Johnson LF, Dubula-Majola V. HIV services in sub-Saharan Africa: The greatest gap is men. Lancet. 2021;397(10290):2130–2132. 10.1016/S0140-6736(21)01163-6 34087108

[43] Bunyasi EW, Coetzee DJ. Relationship between socioeconomic status and HIV infection: Findings from a survey in the Free State and Western Cape Provinces of South Africa. BMJ Open. 2017;7(11):e016232. 10.1136/bmjopen-2017-016232 PMC5719303 29162570

[44] Osler M, Cornell M, Ford N, Hilderbrand K, Goemaere E, Boulle A. Population-wide differentials in HIV service access and outcomes in the Western Cape for men as compared to women, South Africa: 2008 to 2018: A cohort analysis. J Int AIDS Soc. 2020;23(S2):e25530. 10.1002/jia2.25530 32589367 PMC7319137

[45] Statistics South Africa (STATSSA). Mid-year population estimates [homepage on the Internet]. 2021 [cited 2024 Jul 11]. Available from: https://www.statssa.gov.za/publications/P0302/P03022021.pdf

[46] Coetzee L, Cassim N, Da Silva P, Stevens WS, editors. Geographical changes in HIV burden and wellness over the last ten years, using CD4 test results from the National Health Laboratory Service, South Africa. Cape Town: African Society for Laboratory Medicine (ASLM 2023); 2023.

[47] Da Silva MP, Cassim N, Ndlovu S, et al. More than a decade of GeneXpert(®)Mycobacterium tuberculosis/Rifampicin (Ultra) testing in South Africa: Laboratory insights from twenty-three million tests. Diagnostics (Basel). 2023;13(20):3253. 10.3390/diagnostics13203253 37892074 PMC10605857

[48] Fox MP, Bor J, Brennan AT, et al. Estimating retention in HIV care accounting for patient transfers: A national laboratory cohort study in South Africa. PLoS Med. 2018;15(6):1–21. 10.1371/journal.pmed.1002589 PMC5995345 29889844

[49] Moolla H, Davies M, Davies C, et al. The effect of care interruptions on mortality in adults resuming antiretroviral therapy. AIDS. 2024;38(8):1198–1205. 10.1097/QAD.0000000000003859 38814712 PMC11141523

[50] Stevens WS, Cunningham B, Cassim N, Gous N, Scott LE. Cloud-based surveillance, connectivity, and distribution of the GeneXpert analyzers for diagnosis of tuberculosis (TB) and multiple-drug-resistant TB in South Africa. In: Persing DH, Tenover RT, Hayden M, et al editors. Molecular Microbiology. 2016; p. 707–718. 10.1128/9781555819071.ch49

[51] Boulle A, Heekes A, Tiffin N, et al. Data centre profile: The provincial health data centre of the Western Cape Province, South Africa. Int J Popul Data Sci. 2019;4(2):1143. 10.23889/ijpds.v4i2.1143 32935043 PMC7482518

[52] Shearer WT, Rosenblatt HM, Gelman RS, et al. Lymphocyte subsets in healthy children from birth through 18 years of age: The pediatric AIDS clinical trials group P1009 study. J Allergy Clin Immunol. 2003;112(5):973–980. 10.1016/j.jaci.2003.07.003 14610491

